# Could Early Rheumatoid Arthritis Resolve After Periodontitis Treatment Only? Case Report and Review of the Literature

**DOI:** 10.1097/MD.0000000000000195

**Published:** 2014-12-12

**Authors:** Simonetta Salemi, Michela I. Biondo, Chiara Fiorentino, Giuseppe Argento, Michele Paolantonio, Carlo Di Murro, Vito A. Malagnino, Marco Canzoni, Andrea Picchianti Diamanti, Raffaele D’Amelio

**Affiliations:** From the Division of Allergy, Clinical Immunology and Rheumatology (SS, MIB, CF, MC, APD, RD); Division of Radiology (GA), S. Andrea University Hospital, Sapienza University of Rome, Rome; Department of Periodontology (MP, CDM), G. D’Annunzio University; and Department of Endodontics (VAM), G. D’Annunzio University, Chieti-Pescara, Italy.

## Abstract

Rheumatoid arthritis (RA) is an immune-mediated polyarthritis; currently no pathogenic agent has been identified as a disease trigger. A patient with RA, presumably caused by periodontal infection, whose remission has been observed after periodontitis treatment in absence of specific RA therapy, is reported here for the first time, to our knowledge.

A 61-year-old male patient presented migrant arthritis associated with antibodies against citrullinated protein antigens positivity. The clinical features allowed to make RA diagnosis according to the 2010 European League against Rheumatism/American College of Rheumatology RA classification criteria. X-ray of the second upper molar showed chronic apical periodontitis. After its treatment, arthritis remission has been observed in the absence of specific RA therapy.

It has been suggested that periodontitis may have a trigger role in RA pathogenesis. This could be explained by the enzymatic action of *Porphyromonas gingivalis,* probably leading to break tolerance to collagen. The identification and subsequent treatment of periodontitis should therefore be considered pivotal in RA prophylaxis and management.

## INTRODUCTION

Rheumatoid arthritis (RA) is a chronic polyarthritis and is characterized by specific serological alterations, which include the expression of antibodies directed against citrullinated protein antigens (anti-citrullinated protein antibodies [ACPAs]).^1^ In recent years, there have been important advances in RA pathogenesis, together with new diagnostic and therapeutic insights. The identification of a single trigger for RA has been elusive for many years, and multiple studies have failed to identify conclusively an organism singly responsible for the disease. The responsibility of bacterial/viral infections as causes of RA has often been hypothesized; interestingly, an association between periodontitis and RA^2,3^ has been recently described, and different mechanisms have been proposed to clarify this association. Among these, the most convincing evidence is that some bacteria of the oral flora exert a citrullination enzymatic activity that could lead to break tolerance.^4^

A 61-year-old RA patient, in whom diagnosis and subsequent treatment of periodontal infection has led to a resolution of the clinical picture, is reported here. This is, to the best of our knowledge, the first case in which RA has totally been resolved without the intervention of any specific RA treatment.^5–11^

## CASE PRESENTATION

A 61-year-old man was seen in September 2012 at the outpatient Immuno-Rheumatology Clinic of the S. Andrea University Hospital, Rome, Italy, because of the appearance of migrant arthritis 8 weeks before. He reported morning stiffness lasting half an hour. The patient had pain and functional limitation of the right shoulder. The pain persisted at rest and was responsive to etoricoxib, but unresponsive to paracetamol and corticosteroids. He also complained of pain and functional limitation in hands, knees, jaw, and wrists. The pain lasted 24–48 hours.

The patient had a history of recurrent tonsillitis in infancy and a past smoking history. There was no personal or familial history of psoriasis.

Clinical examination showed tenderness and swelling of the second and third metacarpophalangeal (MCP) joints of the left hand and wrists.

Laboratory tests revealed leukocytosis (11,880/μL, neutrophils 75.6%), increase of erythrocyte sedimentation rate ([ESR] 36 mm/h), α2-globulins (1.08 g/dL), C-reactive protein ([CRP] 2.4 mg/dL), and ACPAs positivity (>250 U/mL). Human leukocyte antigen (HLA) haplotype typization revealed the presence of the HLA DRB1∗11, DRB1∗13, and DQB1∗03. Markers of hepatitis B and C viruses, rheumatoid factor (RF), antinuclear antibodies, antimitochondrial antibodies, antistreptolysin O titer, *Treponema pallidum* hemagglutination test, Veneral Disease Research Laboratories, and tuberculin skin test were negative. Urinalysis, urine culture, throat swab culture, and urogenital swab specimens for detection of *Mycoplasma genitalium*, *Ureaplasma urealyticum*, and *Chlamydia trachomatis* were also negative.

**FIGURE 1:**
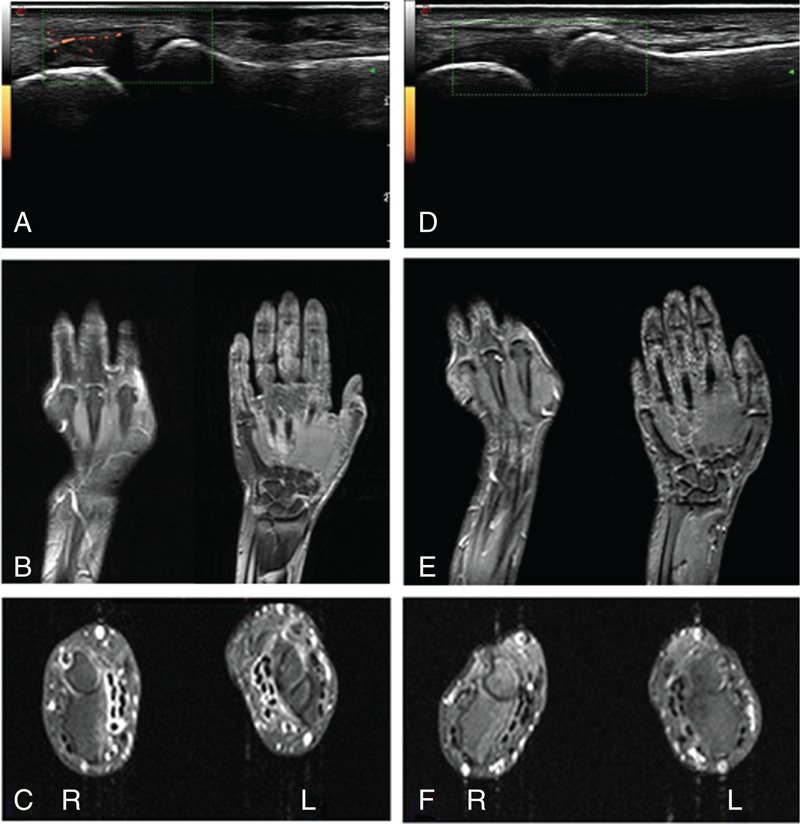
Ultrasonography images of second MCP joint of the left hand and fat-sat gadolinium-enhanced T1-weighted Turbo Spin Echo coronal and transverse magnetic resonance imaging images of left hand and wrists at baseline (A–C) and after periodontal disease treatment (D–F). (A) Moderate active synovitis of the II MCP joint of the left hand (power-doppler grade II). (B) Microerosions of second and third metacarpal head and inflammatory involvement of digital synovial sheaths of third and fourth finger and recessus ulnaris (prestyloideus). (C) Diffuse thickening (enhancement) of sheath of superficial and deep digital flexor tendon and extensor carpi ulnaris tendon of right wrist; less thickening of left wrist. (D) Second MCP joint of the left hand: absence of synovitis (power-doppler negative). (E) Marked reduction of synovial thickening of recessus ulnaris (prestyloideus) and the synovial sheath of third and fourth finger of flexor tendon; persistence of the minimal erosion of third metacarpal head (contrast enhancement). (F) Remarkable reduction of right flexor tendon and extensor carpi ulnaris tendon sheaths thickening. MCP = metacarpophalangeal.

Ultrasonography (US) showed active proliferative synovitis of second and third left MCP joints (gray scale I and power-Doppler signal II) (Figure 1). One and a half month later, magnetic resonance imaging (MRI) of the hands and wrists revealed mild synovitis and bone erosions in the head of the second and third MCP joints of left hand as well as diffuse thickening (enhancement) of sheath of superficial and deep digital flexor tendon and extensor carpi ulnaris tendon of the right wrist, and less thickening of the left wrist (Figure 1).

RA was diagnosed according to the 2010 European League Against Rheumatism/American College of Rheumatology (EULAR/ACR) RA Classification Criteria.^12^ The patient had the involvement of 4 small joints (second and third left MCP and wrists), highly positive ACPAs, high ESR, symptom duration >6 weeks, thus totaling a score of 8/10 (RA diagnosis >6). The Disease Activity Score on 28 joints (DAS28) was 5.7 thus revealing a severe RA activity.^13^

**FIGURE 2:**
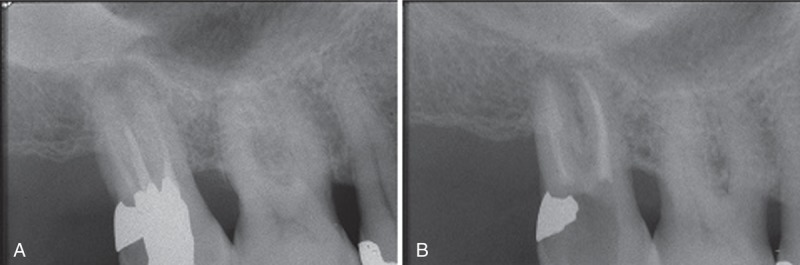
(A) Second upper molar x-ray showed a chronic apical periodontitis on the mesiobuccal root. (B) Bone reconstitution appeared complete in the last second upper molar x-ray.

Nearly 6 weeks later, on the basis of a suspect dental lesion on x-ray, the patient was referred to a dental examination. He reported pain after pressure on tooth 46 as a chief complaint. The second upper molar x-ray showed a chronic apical periodontitis on the mesiobuccal root (see the radiolucency in Figure 2). The tooth had received an inadequate endodontic treatment. An endodontic retreatment was planned in order to remove the root canal system infection, particularly in the mesiobuccal root. The endodontic retreatment was performed in a single visit^14,15^; the root canal preparation was performed with M2 endodontic Ni-Ti instruments (Sweden and Martina, Carrare (Pd), Italy) and the irrigation was carried out with 5.25% sodium hypochlorite; the canals were filled with the Microseal system (Sybronendo, West Collins, Orange, CA).^16^ Simultaneously, the clinical periodontal examination evidenced diffuse signs of gingivitis (60% of gingival sites exhibiting bleeding after probing) and various ≥5-mm-deep periodontal pockets in the upper arch. Periodontal treatment consisted of full-mouth scaling and root planing with accurate oral hygiene instructions. Subsequently, in the right upper quadrant, an open flap debridement was done in periodontal pockets exceeding 5 mm. The patient was then requested to rinse the mouth with chlorhexidine for 2 weeks and he was recalled at 3 and 6 months for clinical control and hygienic prophylaxis. After 6 months, in conjunction with the healing of the endodontic lesion (Figure 2), the periodontal charting showed no probing values >3 mm nor the presence of signs of periodontal infection in the whole mouth.

Approximately 1 month after the dental treatment, the patient reported an improvement of both arthralgias and arthritis. On examination, only the third proximal interphalangeal joint of the right hand was tender and swollen, consequently the DAS28 significantly improved to a level of clinical remission (2.1). Blood tests showed normal values of leukocytes (8600/μL), ESR (5 mm/h), and α2-globulins (0.81 g/dL). Only ACPAs remained elevated (>250 U/mL), while CRP was slightly positive (1.6 mg/dL). MRI of the left hand and wrists revealed synovitis reduction (Figure 1). After 2 months, the persistence of physical well-being was accompanied by CRP negative values. MRI confirmed the preexistent erosions. After 17 months, the well-being persisted and US showed the maintenance of complete recovery (Figure 1), whereas ACPAs were still positive at the same titer.

## DISCUSSION

A complete and long-lasting RA recovery after periodontal treatment in a male patient with RA and periodontitis is reported here. Considering the arthritis migrant trend, the large joint involvement and the apical periodontitis, a reactive arthritis was initially hypothesized. However, the involvement of 4 small joints, confirmed by the presence of synovitis and erosions in MRI and US of the second and third left MCP joints, the ACPAs high positivity, the length of disease > 6 weeks, and the increase of inflammatory indices allowed to fully meet the 2010 EULAR/ACR RA Classification Criteria,^12^ with a total score of 8/10. Although RA with high ACPAs titer has been reported to be characterized by a worse prognosis,^17^ a rapid reversibility of clinical manifestations has instead been observed in this case after periodontitis treatment.

RA is a chronic autoimmune disease characterized by synovial inflammation and pannus formation, eventually leading to cartilage and bone destruction, mediated by the production of autoantibodies including RF and ACPAs.^18^ The citrullination is a post-translational reaction consisting of conversion of arginine into citrulline, which can have important consequences for the protein structure and function, considering that at neutral pH, arginine is positively charged, whereas citrulline is uncharged. This increases the protein hydrophobicity, leading to changes in protein folding. In RA pathogenesis, citrullinated proteins are generated by the activity of specific enzymes, named peptidyl-arginine-deiminases (PAD) type IV, that catalyze the modifications of peptidyl-arginine to peptidyl-citrulline on several self-proteins, including α-enolase, keratin, fibrinogen, fibronectin, collagen, and vimentin. Loss of tolerance to such neoepitopes elicits an ACPAs response that may lead to disease.^17^ ACPAs are considered highly specific for RA, thus suggesting their possible pathogenic role in disease initiation and progression.^19^

Several factors, including infections, have been considered in autoimmunity onset, leading to RA. In the last few years, an epidemiological association between RA and periodontitis has been observed^20^; although the mechanism underlying this association is not clear, periodontitis has been suggested having a role in RA development and progression.^21^ Periodontitis is a highly prevalent chronic inflammatory oral disease,^22^ although the most severe forms are restricted to a limited percentage of population.^23^ It is an opportunistic infection, induced by a limited number of putative periodontopathic microorganisms, occurring in presence of individual predisposition and environmental risk factors,^24^ eventually leading to periodontal tissue destruction and tooth loss, if left untreated. The tissue-destroying process is often caused by Gram-negative anaerobic bacteria, such as *Porphyromonas gingivalis*, *Prevotella intermedia, Tannerella forsythia*, and *Aggregatica bacter-actinomycetem-comitans*.^25^ The high periodontitis prevalence in RA is confirmed by molecular detection of anaerobes and high antibody titers against periodontal bacteria in serum and synovial fluid of RA patients.^26^ A growing interest has been addressed to the correlation between RA and *P gingivalis*-associated periodontitis that represents one of the most investigated pathogens in periodontitis etiology. *P gingivalis* is actually the only known bacterium expressing a PAD enzyme,^27^ responsible for post-transcriptional protein modifications similar to those obtained from human PAD. Although the PAD expressed in *P gingivalis* is quite different from the human variant, it has been demonstrated that it can produce irreversible citrullinated peptides from at least 2 known RA antigens, fibrinogen, and α-enolase.^4^*P gingivalis* infection has also been suggested to be associated with increased risk for RA development.^19^

Periodontitis and RA share common risk factors, mainly including HLA-DRB1 alleles^28^ and smoking^29^; in the patient's medical history, in fact, a previous smoking status was reported. Moreover, both pathological conditions are characterized by an inappropriate inflammatory reaction mediated by immune cells, enzymes, and cytokines, which results in tissue damage. Giving these similarities, it could be postulated that the 2 illnesses may occur simultaneously in individuals with an intrinsic dysregulation of the inflammatory response.^30^

In the case reported here, the successful treatment of periodontal infection has been associated with a progressive improvement of RA clinical manifestations, a gradual resolution of arthritic symptoms, and a gain of US and MRI features. Laboratory tests also returned to normal values and disease activity dramatically improved from high disease activity to remission; only ACPAs remained positive.

The literature reports at least 6 studies in which the treatment of periodontal infection has reduced the severity of active RA, confirming a role of periodontitis not only in initiation but also in disease progression.^5–11^ In particular, Ribeiro et al^5^ observed an improvement of the Health Assessment Questionnaire and a significant reduction of ESR in 26 RA patients with periodontitis after periodontal treatment in 2005. Al Katma et al^6^ were able to confirm the data of clinical and serological RA improvement in 17 RA patients with periodontitis after periodontal treatment, and Ortiz et al^7^ observed a significant improvement of DAS28, with reduction of ESR and circulating tumor necrosis factor (TNF), in 20 RA patients with periodontitis after periodontal treatment. Instead, Pinho et al^8^ observed an improvement after periodontal treatment in 15 RA patients with periodontitis, which was not significant, of RA inflammatory parameters. More recently, Erciyas et al^9^ on 60 RA patients with moderate-to-high and low disease activity, and periodontitis observed a significant improvement of disease activity parameters after periodontal treatment, whereas Okada et al^10^ observed not only a significant improvement of DAS28 in 26 RA patients with periodontitis after periodontal treatment but also a significant reduction of specific antibodies to *P gingivalis* and citrullinated peptides. In all these studies, a specific RA therapy (consisting in the use of disease modifying antirheumatic drugs [DMARDs] and/or TNF inhibitors) has always been part of the treatment, whereas in the present case, a specific therapy has never been carried out. Moreover, what has been observed after periodontal treatment in our case is a complete, long-lasting recovery, not a simple improvement. However, at 17 months from the clinical remission, despite the persistence of well-being and the lack of inflammation, ACPAs still maintain the same high titer, in contrast to what has been reported by Okada et al.^10^ It is possible, therefore, to hypothesize that in selected early RA cases, prompt periodontal infection treatment may induce disease abortion, thus avoiding the development of a chronic and progressive arthritis. This is, to the best of our knowledge, the first case of periodontitis-associated RA healing, through periodontal treatment only, without the use of DMARDs and/or TNF inhibitors.

Limitations of this case report may include possible misclassification. If the diagnosis were performed according to the 1987 ACR criteria, this case could not have been classified as RA, whereas, having been classified according to the more modern and sensitive, but less specific, EULAR/ACR 2010 criteria, the case reported here may easily and convincingly be considered RA. Moreover, the imaging joint pattern was clear-cut and specific for RA. Probably the prompt and complete resolution, never observed before, may even be linked to the lack of a permissive HLA allele.

Although the conclusions from the Joint European Federation of Periodontology/American Academy of Periodontology argue that reports of an epidemiological association between RA and periodontitis offers no absolute evidence,^31^ several observations seem to support such association. The reason underlying this association seems to lie in the ability of periodontal pathogens responsible for gum infection to play a trigger role in RA. A possible mechanism is represented by the *P gingivalis*-induced break tolerance to citrullinated proteins. Oral cavity cleaning should therefore be considered of pivotal importance in RA prophylaxis and management.
